# Relatively low primary drug resistant tuberculosis in southwestern Ethiopia

**DOI:** 10.1186/1756-0500-5-225

**Published:** 2012-05-10

**Authors:** Gemeda Abebe, Ketema Abdissa, Alemseged Abdissa, Ludwig Apers, Mulualem Agonafir, Bouke C de-Jong, Robert Colebunders

**Affiliations:** 1 Department of Medical Laboratory Sciences and Pathology, Jimma University, Jimma, Ethiopia; 2 Department of Epidemiology and Social Medicine, University of Antwerp, Antwerp, Belgium; 3 Department of Clinical Sciences, Institute of Tropical Medicine, Antwerp, Belgium; 4 Infectious and Other Diseases Research Department, Ethiopian Health and Nutrition Research Institute, Addis Ababa, Ethiopia; 5 Mycobacteriology Unit, Department of Microbiology, Institute of Tropical Medicine, Antwerp, Belgium

**Keywords:** Tuberculosis, Drug resistance, Ethiopia, Jimma

## Abstract

**Background:**

The prevalence of drug resistant tuberculosis (TB) in Ethiopia in general, and Jimma area in particular, is not well documented. We conducted a study at Jimma University specialized hospital in southwest Ethiopia among new cases of smear positive TB patients to determine the pattern of resistance to first-line drugs.

**Methods:**

A health institution based cross sectional study was conducted from November 2010 to September 2011. Any newly diagnosed smear positive TB patient 18 years and above was included in the study. Demographic and related data were collected by trained personnel using a pretested structured questionnaire. Mycobacterial drug susceptibility testing (DST) to the first line drugs isoniazid (INH), rifampicin (RIF), ethambutol (EMB) and streptomycin (STM) was performed on cultures using the indirect proportion method. *M. tuberculosis* complex (MTBC) was identified with the Capilia TB-Neo test.

**Results:**

136 patients were enrolled in the study. Resistance to at least one drug was identified in 18.4%. The highest prevalence of resistance to any drug was identified against INH (13.2%) followed by STM (8.1%). There was no statistically significant difference in the proportion of any resistance by sex, age, HIV status and history of being imprisoned. The highest mono resistance was observed against INH (7.4%). Mono resistance to streptomycin was associated with HIV infection (crude OR 15.63, 95%CI: 1.31, 187). Multidrug-resistance TB (MDR-TB) was observed in two patients (1.5%).

**Conclusion:**

Resistance to at least one drug was 18.4% (INH-13.2% and STM-8.1%). STM resistance was associated with HIV positivity. There was relatively low prevalence of MDR-TB yet INH resistance was common around Jimma. The capacity of laboratories for TB culture and DST should be strengthened, in order to correctly manage TB patients and avoid amplification of drug resistance.

## Background

TB is the world’s leading curable cause of infectious diseases mortality, with a disproportionate burden of the disease falling on low and middle income countries. In 2010, there were an estimated 8.8 million incident cases of TB globally. Most of the estimated number of cases occurred in Asia (59%) and Africa (26%) [1]. While most TB cases are in Asia, in Africa the incidence rates are highest, driven by high rates of HIV and malnutrition [2].

One of the aims of ensuring effective management of TB is to minimize the development of drug resistance, which results from inadequate therapy. Surveillance of anti-TB drug resistance is therefore an essential tool for monitoring the effectiveness of TB control programs and improving control efforts. The emergence of drug resistance in recent years has highlighted the importance of an effective control strategy for tuberculosis. The rapid spread of drug resistance, multi-drug resistant TB (MDR-TB) and extensively drug resistant TB (XDR-TB), both in new and in previously treated cases, adds urgency to the need for decisive action to develop and implement control measures [2].

Ethiopia is one of the high burden countries, reflected both in its TB incidence and the estimated rates of MDR TB. However, there has been no reliable estimate on either general prevalence or drug resistance of TB. To date a single TB drug resistance survey was conducted back in 2005 [3]. The laboratory capacity in Ethiopia to diagnose MDR TB is very limited. There was only one public laboratory that became functional two years ago that could perform both MTB culture and drug resistance testing. As a result national estimates were based on incomplete data that suffer from representativeness since the reporting system is poorly developed, diagnostic criteria are usually non-standardized and many MDR cases go undetected [4].

WHO estimates for Ethiopia indicates that the rate of MDR-TB is 1.6% for new cases and 12% for retreatment cases [1]. Reports from different parts of Ethiopia suggest that the rate of drug resistant TB is highly variable across the country [5-9]. To date there is however no report on anti-tuberculosis drug resistance from the southwest part of Ethiopia in general and the Jimma area in particular. The aim of the present study was to determine the susceptibility to STM, INH, RIF and EMB of *Mycobacterium tuberculosis* complex strains isolated from patients newly diagnosed with pulmonary tuberculosis at Jimma University Specialized hospital.

## Methods

### Study setting

The study was conducted at Jimma University Specialized hospital, located in Jimma town, southwest Oromia, Ethiopia, at a distance of 355 Km from the capital city Addis Ababa. The total population of Jimma zone according to the 2005 census report is 2,773,730, of whom 1,382,460 (49.8%) were males. According to this report 340,666 (12.3%) of the Jimma population live in an urban environment. With an estimated area of 18,412.54 square kilometers, the population density was 151 people per square kilometer.

### Study design and period

In this cross sectional study we enrolled all consenting smear-positive new cases of TB patients aged ≥ 18 years. Three consecutive sputum samples (spot, early morning and spot) were taken from each patient according the Ethiopian National TB and Leprosy guidelines. Only the samples smear positive for acid fast bacilli by Ziehl Neelsen smear microscopy were further processed by culture (one per patient). Samples were stored at the Jimma University Mycobacteriology laboratory at 4°C and were cultured within 48 hours. TB patients enrolled in the study completed a questionnaire containing demographic information, history of anti-TB treatment, history of contact with chronic coughers, use of injectable drugs, history of diabetes, alcohol usage and history of imprisonment. The HIV status was collected from records. At Jimma University specialized hospital voluntary counseling and testing for HIV was routinely offered to all TB patients, as recommended by WHO in countries with a generalized HIV epidemic [10]. The study was conducted from November 2010 to September 2011.

### Sample size

The number of new smear positive cases to be included in this study was calculated according to the sampling method described in "*Guidelines for surveillance of drug resistance in tuberculosis"* developed by WHO/IUATLD [11], using the following parameters: the number of smear positive cases registered at Jimma University specialized hospital during 2008/2009 was 495, the resistance to rifampicin in new TB cases from a previous study in Ethiopia was 1.2% [5] and the degree of desired precision was 1.5%. To compensate for expected losses the calculated sample size was increased by 15%, resulting in a total sample size of 156 patients.

### Sputum sample processing

Good quality specimens (inspected visually) with enough volume (2.5-10 ml) were processed by the standard N-acetyl L-cysteine (NALC)-NaOH method [12] and concentrated at 3000 × g for 20 minutes. The sediment was reconstituted to 2.5 ml with phosphate buffer (pH 6.8) to make the suspensions for the smears and cultures.

### Culture and identification

One sputum sample per patient was cultured for the presence of *M. tuberculosis* complex by use of either Löwenstein-Jensen (LJ) slants (November 2010 to April 2011) or the BACTEC MGIT 960 system (Becton Dickinson Microbiology systems, Sparks, MD, USA) (May to September 2011), according to the manufacturers recommendations [13]. LJ slants were inoculated with the sediment, incubated at 37°C and examined weekly for growth. Cultures were considered negative when no colonies were seen after 8 weeks incubation. In case of MGIT 960, the MGIT tubes (7 ml) were inoculated with the processed specimen, the tubes were incubated at 37 ^0^ C in the BACTEC MGIT 960 instrument and were monitored automatically every 60 minutes for increase in fluorescence [13]. The cultures were tested until positive or for 6 weeks. The isolates from either LJ or MGIT 960 tubes were confirmed to be MTB complex by combination of microscopic observation for serpentine cord formation and testing with Capilia TB-Neo (Tauns Laboratories, Inc, Shizuoka, Japan).

### Drug susceptibility testing

All *M. tuberculosis* complex strains as determined by cord formation and Capilia test were subjected to susceptibility testing to the first line anti-tuberculosis drugs on either LJ (November 2010 to April 2011) or MGIT 960 tubes (May to September 2011). The indirect proportion method on LJ media was performed at the following final drug concentrations: STM at 4 μg/ml, INH at 0.2 μg/ml, RPM at 40 μg/ml and EMB at 2 μg/ml. The slopes were incubated at 37^0^ C and read daily for one week for the possibility of contamination and growth of fast growing mycobacteria, and then read at 28 days and at 42 days. Drug resistance was defined as growth on a drug containing medium greater than or equal to 1% of that recorded on the drug-free control medium of the same experiment. DST using MGIT 960 followed standard procedures according to the manufacturer’s recommendation [13]. Final drug concentrations were 1.0 μg/ml for STM, 0.1 μg/ml for INH, 1.0 μg/ml for RMP and 5.0 μg/ml for EMB. The results were automatically interpreted by the BACTEC MGIT 960 instrument and reported as either susceptible or resistant.

### Quality control and purity checks

The reference strain of *M. tuberculosis* H37Rv (ATCC 27294), which is susceptible to all standard anti-tuberculosis drugs, was used in each batch of media prepared with drugs. All bacterial suspensions used for DST in MGIT 960 were checked for purity on blood agar and sabouraud dextrose agar. In every DST step H37Rv (ATCC 27294) was used as susceptible control.

### Statistical analysis

Data were entered and analyzed by SPSS version 15.0 statistical software (SPSS Inc. Chicago, 2007). Descriptive analysis was done to depict the socio-demographic variables and proportion of drug resistant TB. Univariate logistic regression test was used to evaluate the association between drug resistance and socio-demographic status, HIV infection and a history of imprisonment. P value less than or equal to 0.05 was considered statistically significant.

### Ethical consideration

The study protocol was approved by the ethical review committees of Jimma University, Ministry of Science and Technology, Ethiopia and University of Antwerp, Belgium. Written informed consent was obtained from the study participants. The DST results were communicated to the treating physicians. MDR-TB cases were referred to a specialized central MDR-TB clinic in Addis Ababa to start treatment.

## Results

During the study period at Jimma University specialized hospital a total of 176 sputum smear positive TB patients were registered of whom 156 new cases of sputum smear positive TB patients were enrolled. Twenty patients were excluded based on previous history of anti-TB treatment for more than one month. The 156 participants provided 468 sputum samples for microscopy. One hundred and fifty-six smear positive sputum samples (one per patient) were cultured, which grew *M. tuberculosis* in samples from 136 (87.2%) patients, while cultures from 12(7.7%) patients were contaminated and 8 (5.1%) did not grow. Samples that were contaminated or failed to grow were excluded from further analysis. Of the 136 patients with viable isolates, the majority (57.4%) were males. Thirteen (9.6%) were HIV seropositive, of whom 5 were receiving antiretroviral therapy. The age of the study subjects ranged from 18 to 64 years with median age of 26.0 (± 10.7) years (Table 1).

**Table 1:** Characteristics of study participants by age, sex and HIV status

	**Sex**		**HIV status**		
**Age**	**Male** **n(%)**	**Female** **n(%)**	**Positive n(%)**	**Negative n(%)**	**Unknown n(%)**
18-24	33 (62.3)	20(37.7)	2(3.8)	38(71.7)	13(24.5)
25-34	28 (54.9)	23 (45.1)	9(17.6)	30(58.8)	12(23.5)
35-44	7(41.2)	10(58.8)	1(5.9)	11(64.7)	5(29.4)
> = 45	10(66.7)	5(33.3)	1(6.7)	8(53.3)	6(40.0)
Total	78(57.4)	58(42.6)	13(9.6)	87(64.0)	36(26.5)

All the 136 isolates were identified as *M. tuberculosis* complex. Among these, 111 (81.6%) were susceptible to all the drugs tested, while 25 (18.4%) were resistant to at least one drug. Resistance to more than one drug (excluding the combination of INH and RIF) was observed in 6(4.4%) patient isolates (Table 2). When each drug was considered separately, the highest rates of resistance (single or in combination) were observed for INH (13.2%) and STM (8.1%), followed by EMB (5.2%) and RMP (2.2%). A total of 4 isolates (2.9%) were resistant to two drugs only, 2 (1.5%) to three drugs only and 2(1.5%) to four drugs. Highest mono resistance was observed against INH (7.4%). Two (1.5%) MDR-TB strains (resistant to both INH and RIF) were found.

**Table 2:** Resistance pattern to first-line drugs (n = 136)

**Resistance**	**N(%)**	**95% CI**
Any resistance	25(18.4)	12.8-25.7
Any STM	11(8.1)	4.6-13.9
Any INH	18(13.2)	8.5-20.0
Any RIF	3(2.2)	0.8-6.3
Any EMB	7(5.2)	2.5-10.2
**Mono resistance**		
STM	4(2.9)	1.2-7.3
INH	10(7.4)	4.0-13.0
RIF	1(0.7)	0.1-4.1
EMB	2(1.5)	0.4-5.2
**INH and RIF Resistances (MDR)**		
INH + RIF only	0	
INH + RIF + EMB only	0	
INH + RIF + STM only	0	
INH + RIF + EMB + STM only	2(1.5)	0.4-5.2
**INH and other resistances**		
INH + EMB only	1(0.7)	0.1-4.1
INH + STM only	3(2.2)	0.8-6.3
INH + EMB + STM only	2(1.5)	0.4-5.2
**RIF and other resistances**		
RIF + EMB only	0	
RIF + STM only	0	
RIF + EMB + STM only	0	
**Poly Resistance**	6(4.4)	2.0-9.3

RIF = Rifampicin,INH = Isoniazid, EMB = Ethambuthol, STM = Streptomycin, MDR = multi-drug resistance, CI = Confidence interval.

Of the 13 HIV-infected patients, 3 had isolates resistant to at least one of the four drugs but none of them were MDR-TB**.** There was no statistically significant difference in the proportion of any resistance by sex, age, HIV status and history of being imprisoned (Table 3) with the exception of mono resistance to streptomycin, which was associated with HIV infection (crude OR 15.63, 95%CI: 1.31, 187) (data not shown).

**Table 3:** Univariable analysis for risk factors for any anti-TB drug resistance

**Characteristic**	**Any resistance**		COR, 95% CI*	**P value**
	**Yes n(%)**	**No n(%)**		
**Sex**				
Male	15(19.2)	63(80.8)	1	0.77
Female	10(17.2)	48(82.8)	1.14(0.47, 2.77)	
**Age**				
18-24	7(13.2)	46(86.8)	1	0.20
25-34	13(25.5)	38(74.5)	0.45(0.16, 1.23)	
35-44	1(5.9)	16(94.1)	2.44(0.28, 21.35)	
> = 45	4(26.7)	11(73.3)	0.42(0.10, 1.69)	
**HIV infection**				
Positive	4(23.1)	9(76.9)	1	0.25
Negative	15(14.9)	72(85.1)	2.13(0.58, 7.85)	
**History of being in prison**				
Yes	2(22.2)	7(77.8)	1	0.76
No	23(15.7)	104(85.0)	1.29(0.25, 6.63)	

COR = Crude odds ratio, CI = confidence interval.

## Discussion

The re-emergence of TB as a global health problem over the past two decades, together with the escalating HIV epidemic and increasing anti-tuberculosis drug resistance, represents a serious problem for TB control. Compounded with poorly developed diagnostic facilities, information on drug resistance from Ethiopia is inadequate. We therefore performed a survey on primary anti-tuberculosis resistance, an indicator of the NTP’s performance in controlling the spread of drug resistance.

The overall observed primary resistance rate of 18.4% is lower than the 26.9% rate reported in the Ethiopian national survey performed in 2005 [3] and the study in the eastern part of the country (32.5%) [8], but is close to the rate reported in central (18.2%) [14] and Northwest part of the country (15.3%) [15]. Our study is single institution based, thus cannot be compared with the Ethiopian national survey. However, our data illustrates the heterogeneity of TB drug resistance in Ethiopia. Most of our study participants were from a rural area. The study from the eastern part of Ethiopia was conducted before 1997 when the DOTS program was not fully implemented in the area. Our result is higher than the sub regional study report from Zimbabwe (3.7%) [16]. Compared with recent national surveys in other regions of Africa our figures are higher than those from Rwanda (10.4%), Tanzania (6.8%) and Botswana (10.4%), but similar with the one from Côte d'Ivoire (23.8%) [3].

Most frequent resistance in our study was against INH (13.2%) followed by STM (8.1%). Strains that were resistant to only one drug were mainly resistant to INH (7.4%) or STM (2.9%). The lowest resistance was against RIF (2.2%). Other studies from the region have reported similar findings [5,15,17,18]. This high INH resistance has implications for both treatment and prevention of TB. First, INH is an integral component of standard DOTS regimen. Secondly, in high INH resistance settings the effectiveness of INH preventive therapy may be compromised. Observations from different studies indicate that resistance to both INH and STM increases the risk of selection for MDR-TB during the intensive phase of treatment [17,19]. In some strains of *M. tuberculosis* mono resistance to STM was related to development of MDR [20]. Since the population structure of the *M. tuberculosis* complex in Ethiopia is not well documented, molecular characterization of strains from the region is recommended.

The clinical management of TB is complicated when isolates are resistant to both INH and RIF (MDR). There are few drugs available to treat MDR-TB. It takes longer (up to 24 months) to treat such cases and the drugs are more toxic. The treatment success rate is generally poor compared with drug susceptible TB [21-23]. The proportion of MDR-TB in our study is consistent with the national survey findings [3], WHO estimate for Ethiopia in 2010 [1] and the recent report from Addis Ababa [5] but higher than the report from northwest part of Ethiopia [15]. The two MDR-TB strains in our study were resistant to all the four drugs tested. No MDR-TB strain was isolated from HIV seropositive patients. The heterogeneous nature of MDR-TB in Ethiopia necessitates regular surveys. To this effect, the laboratory capacity strengthening should be promoted so that systematic follow-up of trends could be possible through regular testing of retreatment cases. Currently, the laboratory capacity in the country is inadequate. WHO recommends 1 TB culture facility with DST for 5 million people. Currently in Ethiopia there is only one such public facility per 50 million people [1].

Our study was not without pitfall. The major limitation was that we used only Capilia and serpentine cord formation to identify *Mycobacterium tuberculosis* complex. These two methods are not confirmatory. Both false positive and false negative results do occur with Capilia tests [24,25].

## Conclusion

Resistance to at least one drug was 18.4% (INH-13.2% and STM-8.1%). STM resistance was associated with HIV positivity. There was a relatively low MDR-TB but high INH resistance rate around Jimma. The capacity strengthening of laboratories for TB culture and DST should be promoted.

## Competing interests

The authors declared that there is no competing interest.

## Authors’ contribution

**GA** was involved in the conception and design of the study, coordinated the lab work, analyzed the data and drafted the manuscript. **KA** was involved in design of the study, lab works and reviewed the article. **AA** was involved in the design and reviewed the article**. MA** provided reagents and reviewed the article. **BCdJ** critically reviewed the article. **LA** and **RC** participated in the design of the study and critically reviewed the article.
